# Sunflower inflorescences absorb maximum light energy if they face east and afternoons are cloudier than mornings

**DOI:** 10.1038/s41598-020-78243-z

**Published:** 2020-12-09

**Authors:** Gábor Horváth, Judit Slíz-Balogh, Ákos Horváth, Ádám Egri, Balázs Virágh, Dániel Horváth, Imre M. Jánosi

**Affiliations:** 1 Environmental Optics Laboratory, Department of Biological Physics, ELTE Eötvös Loránd University, Pázmány sétány 1, 1117 Budapest, Hungary; 2 Department of Astronomy, ELTE Eötvös Loránd University, Pázmány sétány 1, 1117 Budapest, Hungary; 3 Meteorological Institute, Universität Hamburg, Bundesstrasse 55, 20146 Hamburg, Germany; 4 Danube Research Institute, MTA Centre for Ecological Research, Karolina út 29-31, 1113 Budapest, Hungary; 5 Department of Physics of Complex Systems, ELTE Eötvös Loránd University, Pázmány sétány 1, 1117 Budapest, Hungary

**Keywords:** Ecology, Plant sciences, Environmental sciences, Optics and photonics

## Abstract

The mature inflorescence of sunflowers (*Helianthus annuus*) orients eastward after its anthesis (the flowering period, especially the maturing of the stamens), from which point it no longer tracks the Sun. Although several hypothetical explanations have been proposed for the ecological functions of this east facing, none have been tested. Here we propose an atmospheric-optical explanation. Using (i) astronomical data of the celestial motion of the Sun, (ii) meteorological data of diurnal cloudiness for Boone County located in the region from which domesticated sunflowers originate, (iii) time-dependent elevation angle of mature sunflower heads, and (iv) absorption spectra of the inflorescence and the back of heads, we computed the light energy absorbed separately by the inflorescence and the back between anthesis and senescence. We found that the inflorescences facing east absorb the maximum radiation, being advantageous for seed production and maturation, furthermore west facing would be more advantageous than south facing. The reason for these is that afternoons are cloudier than mornings in the cultivation areas of sunflowers. Since the photosynthesizing green back of mature heads absorbs maximal energy when the inflorescence faces west, maximizing the energy absorbed by the back cannot explain the east facing of inflorescences. The same results were obtained for central Italy and Hungary, where mornings are also less cloudy than afternoons. In contrast, in south Sweden, where mornings are cloudier than afternoons, west-facing mature inflorescences would absorb the maximum light energy. We suggest that the domesticated *Helianthus annuus* developed an easterly final orientation of its mature inflorescence, because it evolved in a region with cloudier afternoons.

## Introduction

The leaves and the immature non-flowering head of common sunflowers (*Helianthus annuus*) continuously change their orientation so that they always face approximately normal to the incoming solar radiation throughout the day^1^. At sunset the immature head faces nearly west, while at night it reorients and turns nearly east well before sunrise^2,3^. Sunflower anthesis (the period of flowering, especially the maturing of the stamens) begins approximately on the 60th day after sowing, when the inflorescence begins to open, immature ray flowers become visible, flowers are not heliotropic anymore, and usually get locked facing east/northeast^4^. Leaf expansion also ceases at anthesis, when the sunflower begins investing its resources in seed production in its flowering head. Solar tracking of sunflower inflorescences slows down and stops by anthesis due to a putative molecular signal and/or structural change^5^. The head contributes more than 25% of the whole-plant light absorption at maturity^6^. After anthesis, the fruiting head bends gradually more and more downwards due to its increasing weight^7^, and the heliotropism of sunflower leaves continues with a dampened amplitude^2^.

Besides the domesticated and wild *H. annuus* sunflowers, some wild relatives also have a daily east to west movement^8,9^. Many other taxa have similar forms of floral heliotropism, including *Chrozophora tinctoria* (Euphobiaceae), *Xanthium strumarium* (Asteraceae), and diverse arctic and alpine species, for example^10–13^.

There are several non-mutually exclusive hypothetical explanations for the ecological functions of the final eastward orientation of the inflorescence of mature sunflower heads:

1. Non-skyward orientation may be adaptive, because the narrower perch could reduce seed depredation by birds^14^. This explanation, however, is directionally non-selective and holds true for any azimuth angle.
2. The easterly direction of the flowering heads may have the advantage of reducing the heat load at noon^4,15^. However, a fixed westerly orientation has the same advantage.
3. The eastward orientation permits greater reception of solar radiation in the early morning, which may speed up drying of morning dew and thus could reduce the opportunity for fungal attack^4^. This idea has not been tested experimentally.
4. The eastward orientation may promote sunflower attractiveness to pollinators through increased morning interception of solar radiation, coincident with the daily timing of anther emergence and pollen presentation^16^. In field experiments Atamian et al.^17^ showed that the surface temperature of sunflower blooms contributes to, but does not solely determine, the differential attractiveness of east- and west-facing flowers to pollinators.
5. Eastward orientation could reduce heat load especially during afternoon periods of high irradiance^14^. Keeping cooler floret temperatures may boost yield or fitness by preventing reductions in pollen viability and fertilization (pollen sterility increases at temperatures > 30 °C) or by improving grain filling during seed development and set^14,18,19^. However, in afternoons the air temperature, rather than the solar irradiance, is usually higher than in mornings^20^.
6. Higher head temperatures result in more rapid seed maturation and reduced grain filling^18^. The surface temperature of eastward oriented sunflower heads is 3–8 °C lower than that of heads artificially facing skyward at midday^4,16^. However, due to symmetry, the same is also true for westward oriented sunflower heads.

Although many physiological details of sunflower heliotropism and nocturnal reorientation have been revealed^2,5,7–9,17,21–25^, the ecological functions of the eastward orientation of mature heads are still unresolved. In this work we propose an atmospheric-optical explanation for the final easterly orientation of sunflower inflorescences. According to our hypothesis, the east facing of the flowering head maximizes the solar and sky radiation absorbed by the inflorescence, which is advantageous for seed production and maturation. Using numerical calculations with astronomical (solar movement in the sky), meteorological (diurnal change of cloudiness) and physiological (absorption spectra and time-dependent elevation angle of sunflower heads) input data, we show that in the region from which domesticated sunflowers originate (e.g. Boone County in eastern North America, rather than Mexico as has earlier been hypothesized^26^) sunflower inflorescences facing east, rather than west or south, absorb the maximum amount of radiation, because afternoons are usually cloudier than mornings. This complementary explanation does not invalidate the earlier hypotheses emphasizing the ecological significance of eastward orientation of mature sunflower inflorescences.

## Results

### Solar path in Boone County

**Figure 1:**
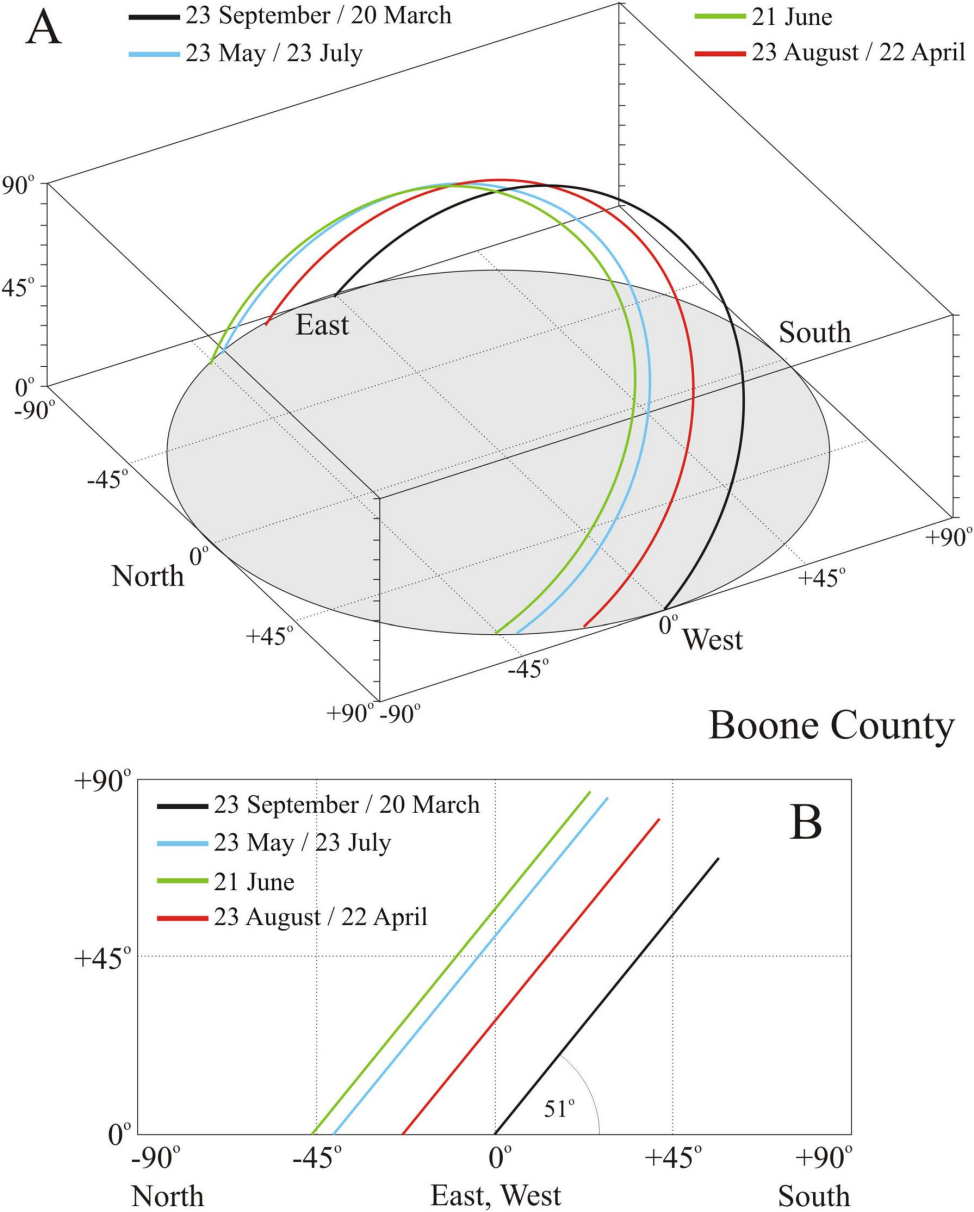
Calculated monthly paths of the Sun on the sky-dome in Boone County (Kentucky, USA, 39° N, − 84.75° E) on 23 May, 21 June, 23 September, and 23 August. Local time is Greenwich Mean Time (GMT) − 5 h. (**A**) *x*–*y*–*z* perspective view. (**B**) *y*–*z* side view.

One of the most important input data for our calculations were the solar paths across the sky in Boone County (Kentucky, USA, 39° N, − 84.75° E) in July, August, and September (Fig. 1). The sun’s orbit is a circle, the tilt angle of which is 90°–39° = 51° from the horizontal in Boone County. Table 1 contains the earliest time of sunrise, the latest time of sunset and the temporal range of solar noon in July, August, and September in Boone County. Between 21 December and 21 June the direction of sunrise moves from southeast through east to northeast, while the direction of sunset moves from southwest through west to northwest. On 21 June these directions reverse and move backwards until 21 December. The elevation angle of solar culmination increases from 27.6°, reaches its maximum of 74.5° on 21 June, then decreases until 21 December. The dates of the spring and autumn equinox and solstice change slightly from year to year as follows: 20–21 March, 20–22 June, 22–23 September and 21–22 December.

**Table 1:** The earliest sunrise, the temporal range of solar noon, and the latest sunset (standard local time) in July, August, and September in Boone County (39.0° N, − 84.75° E).

Month	Earliest sunrise	Solar noon	Latest sunset
July	5 h 17 min	12 h 42–45 min	20 h 8 min
August	5 h 39 min	12 h 39–45 min	19 h 50 min
September	6 h 7 min	12 h 28–38 min	19 h 9 min

### Diurnal cloud patterns

**Figure 2:**
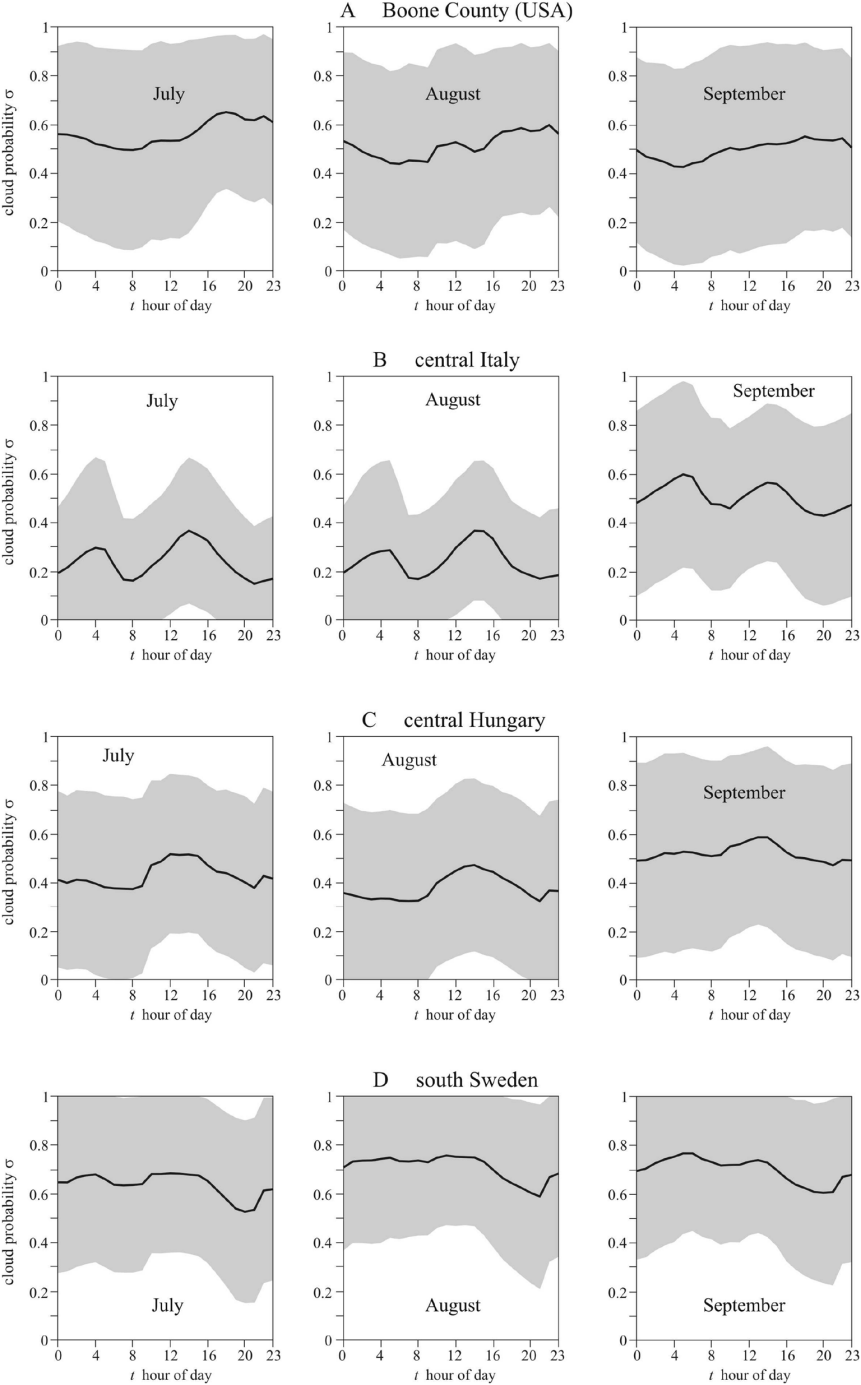
Cloudiness in Boone County and three European regions where sunflowers are cultivated. Diurnal mean probability σ(*t*) (black solid curve) ± standard deviation (grey shading) of cloudy sky in (**A**) Boone County (Kentucky, USA, 39° N, − 84.75° E), (**B**) central Italy (41.0° N, 15.0° E), (**C**) central Hungary (47.0° N, 19.0° E), and (**D**) south Sweden (58.0° North, 13.0° East) averaged for every hour and every day in July, August, and September from ERA5 Total Cloud Cover data (period: 2009.01.01–2018.12.31, temporal resolution: 1 h, spatial resolution: 0.25° × 0.25° ≈ 27 km × 27 km). The local time *t* ranges from 0 to 24 h (0–1, 1–2, 2–3,…, 23–24 h) with 920 data points in every hour bin.

Figure 2 shows the diurnal cloud probability σ(*t*) in July, August, and September in Boone County (Kentucky, USA, 39° N, − 84.75° E, Fig. 2A), central Italy (41.0° N, 15.0° E, Fig. 2B), central Hungary (47.0° N, 19.0° E, Fig. 2C), and south Sweden (58.0° North, 13.0° East, Fig. 2D). In Boone County, central Italy and central Hungary the summer afternoons (between solar noon and sunset) are on average cloudier than the mornings (from sunrise to solar noon), that is σ_morning_ < σ_afternoon_, while in south Sweden the summer mornings are on average cloudier than the afternoons, that is σ_morning_ > σ_afternoon_.

### Elevation angle of mature sunflower heads versus time

**Figure 3:**
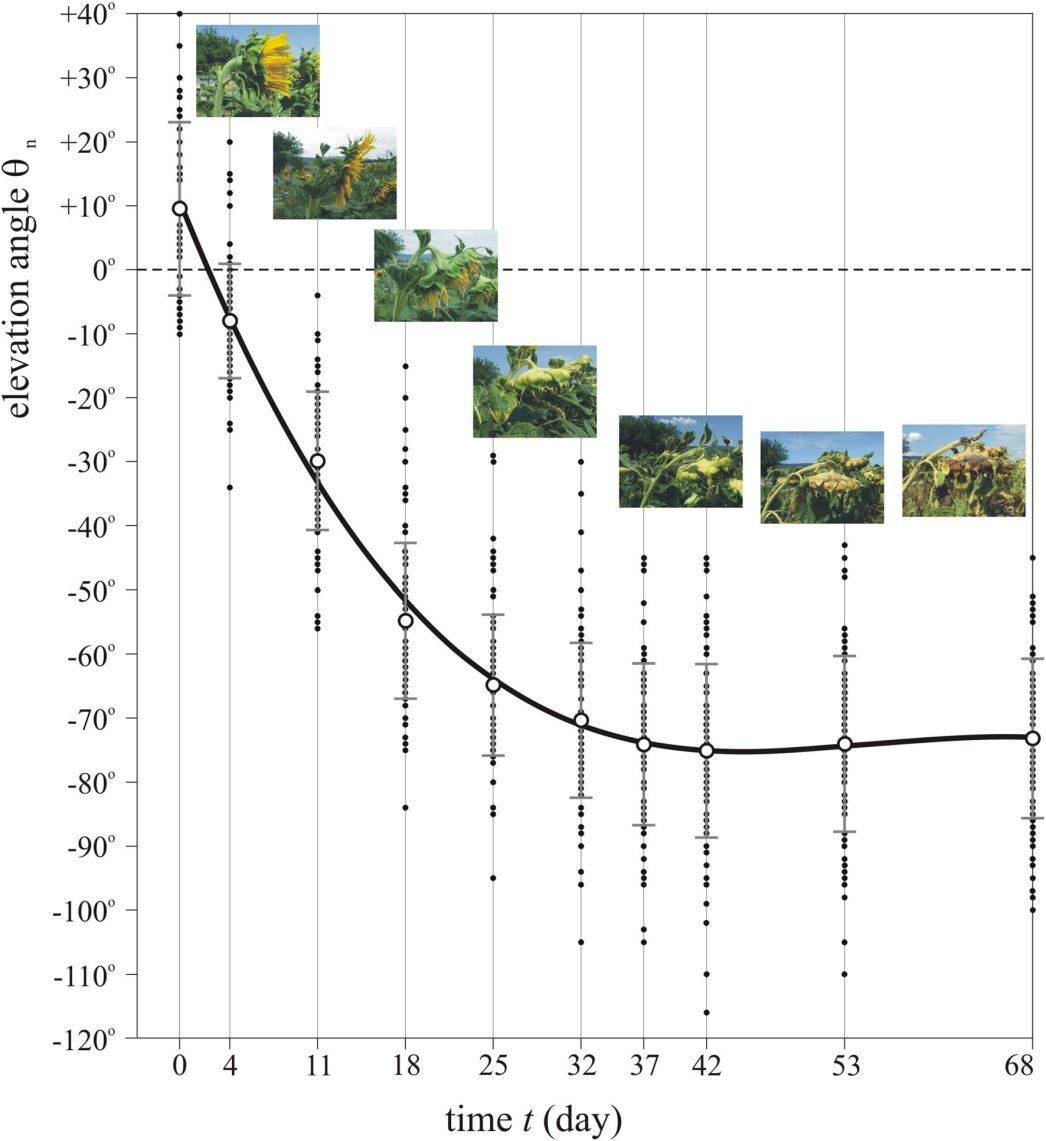
Elevation angle of mature sunflower heads versus time. Elevation angles θ_n_ of the normal vector of the mature head of 100 sunflowers as a function of time *t* given in day after anthesis. Small black dots show the individual θ_n_-values relative to the horizontal, while the large circles and vertical bars represent the mean and standard deviation. The solid black curve is the cubic function θ_n_(*t*) = *at*^3^ + *bt*^2^ + *ct* + *d* with *a* =  − 5.65747017 × 10^–4^, *b* = 9.34138559 × 10^–2^, *c* =  − 4.96385037, *d* = 10.4936951 fitted to the data points. The insets show typical sunflower heads (with increasing age from left to right) photographed by Gábor Horváth from the side. The horizontal positions of these pictures do not correspond exactly to the time represented by the vertical lines.

Figure 3 displays the elevation angles θ_n_ of the normal vector of 100 mature sunflower inflorescences as a function of time *t*. According to the numerical fit to the measured data, the elevation angle θ_n_(*t*) versus time is the following:

1 $$\begin{array}{*{20}l} {\theta _{{\text{n}}} \left( t \right){\mkern 1mu} = at^{3} + bt^{2} + ct + d,} \hfill \\ {a = {\mkern 1mu} - 5.65747017 \times 10^{{ - 4}} ,b = 9.34138559 \times 10^{{ - 2}} ,c = {\mkern 1mu} - 4.96385037,d = 10.4936951,} \hfill \\ \end{array}$$

where *t* is given in day. In the first 3 weeks after anthesis the average θ_n_ decreases steeply from 10°, reaches its minimum at − 75° on the 42nd day, and finally increases slightly to − 72°. The steady decrease of θ_n_ is mainly due to the gradual increase of the head’s weight. Although the final small increase of θ_n_ is statistically non-significant because of the high standard deviations, it is a real biological effect: during necrosis of the curved end of the stem at the head, the stem’s upper side dries quicker than its lower side (and thus the upper stem length decreases faster than the lower one), because the former receives more direct sunlight than the latter.

### Absorption spectra of the inflorescence and the back of mature sunflower heads

**Figure 4:**
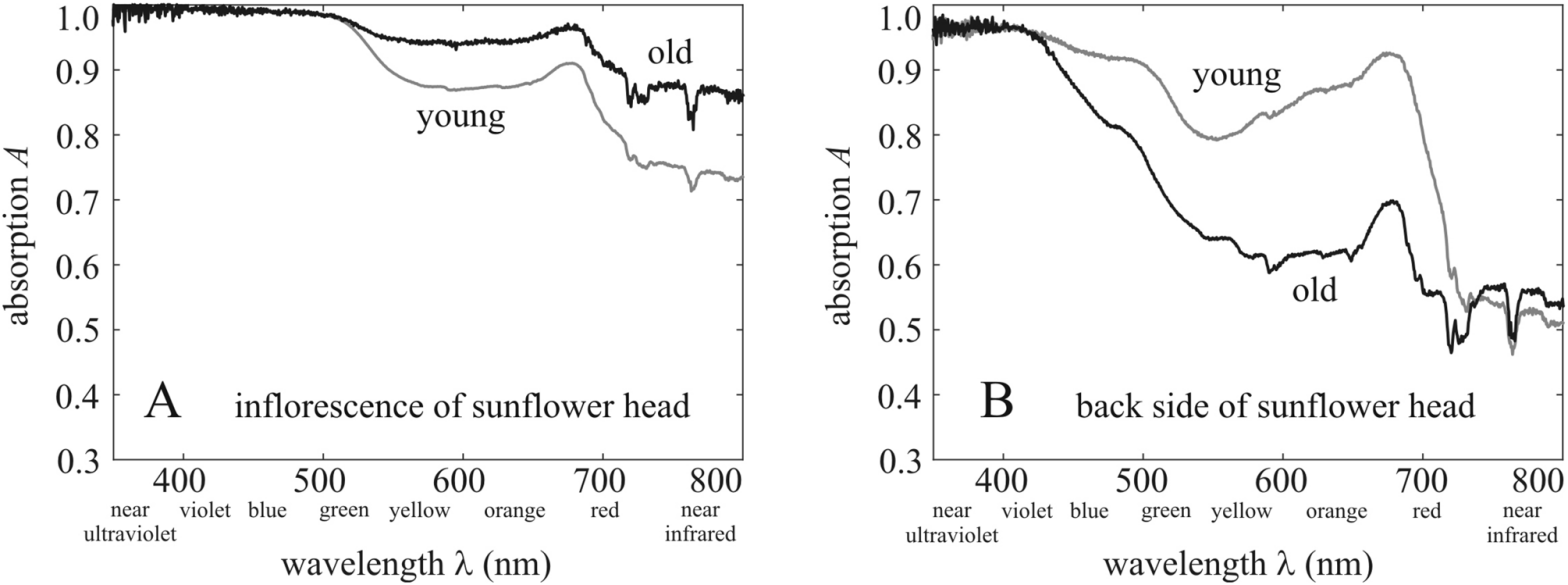
Absorption spectra of sunflower heads. Average absorption spectra *A*(λ) of young (2 weeks) and old (4 weeks) inflorescence (**A**) and back (**B**) of sunflower heads, where λ is the wavelength of light.

Figure 4 illustrates the average absorption spectra 0 ≤ *A*(λ) ≤ 1 of young (2 weeks after anthesis) and old (4 weeks after anthesis) inflorescence and back of the sunflower head measured in the field under overcast conditions. For both young and old inflorescences, light absorption was weaker in the red and near infrared (NIR) spectral ranges than in the blue, violet, and near ultraviolet (NUV) ranges (Fig. 4A). In the green, red, and NIR parts of the spectrum the yellow coloured younger inflorescences had a smaller absorbance than the orange coloured older ones. The absorption of both young and old inflorescences had a wide primary maximum (*A* ≈ 1) spanning the NUV, violet, and blue ranges, and a narrower secondary maximum around 680 nm.

The shape of the absorption spectra for the back of sunflower heads (Fig. 4B) is similar to the inflorescences with two main differences: Firstly, light absorption of the back is weaker at almost every wavelength than that of the inflorescence (Fig. 4A). Secondly, the yellowish-green older backs have weaker absorbance than the green younger backs, apart from the NUV. The absorption of both young and old backs has a wide primary maximum (*A* ≈ 1) in the NUV and violet, and a narrower secondary maximum around 680 nm.

### Energy absorbed by the inflorescence and the back of sunflower heads

**Figure 5:**
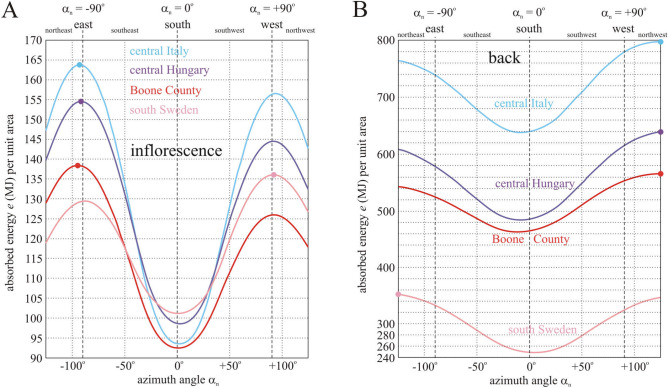
Light energy absorbed by a sunflower head versus its azimuth direction. Total energy *e* per unit area absorbed by the inflorescence (**A**) and the back (**B**) of a mature sunflower head between anthesis (1 July) and senescence (7 September) as a function of the azimuth angle α_n_ of the head’s normal vector computed for Boone County, central Italy, central Hungary, and south Sweden. The primary maxima of curves are indicated by dots.

Let us first consider Boone County representing the region from which domesticated sunflowers originate. Figure 5 exhibits the total light energy *e*_total_ per unit area absorbed by the inflorescence and the back of a mature sunflower head between anthesis (1 July) and senescence (7 September) as a function of the azimuth angle α_n_ of the head’s normal vector. Table 2 presents the primary and secondary maxima and the minimum of *e*_inflorescence_ and *e*_back_ as well as *e*_sum_ = *e*_inflorescence_ + *e*_back_. The total energy absorbed by the back is 4–5 times larger than that absorbed by the inflorescence. According to Fig. 5A and Table 2, the inflorescence absorbs maximal energy at α_max1_ =  − 94°, which is almost equal to the east direction (α_east_ =  − 90°). The function *e*(α_n_) of the inflorescence has a secondary maximum at α_max2_ =  + 92° being approximately the same as the west direction (α_west_ =  + 90°). The minimum of *e*(α_n_) is at α_min_ =  + 1° coinciding practically with the south direction (α_south_ = 0°). An inflorescence facing east absorbs *e*_max1_/*e*_max2_ = 1.1 and *e*_max1_/*e*_min_ = 1.5 times more energy than that facing west and south (Table 2). In contrast, an inflorescence facing west instead of south absorbs *e*_max2_/*e*_min_ = 1.36 times more energy.

**Table 2:** Total energies per unit area *e*_inflo_ and *e*_back_ absorbed by the inflorescence and the back of a mature sunflower head between anthesis (1 July) and senescence (7 September) in Boone County, central Italy, central Hungary, and south Sweden.

	Boone County		Central Italy		Central Hungary		South Sweden	
	*e*_inflo_	*e*_back_	*e*_inflo_	*e*_back_	*e*_inflo_	*e*_back_	*e*_inflo_	*e*_back_
*e*_max1_ (MJ)	138.4	566.0	163.7	797.6	154.5	638.1	136.1	352.0
*e*_max2_ (MJ)	125.9	543.6	156.5	763.9	144.5	608.6	129.4	346.6
*e*_min_ (MJ)	92.5	463.5	93.5	637.8	98.6	483.7	101.2	249.7
*e*_max1_/*e*_max2_	1.10	1.04	1.05	1.04	1.07	1.05	1.05	1.02
*e*_max1_/*e*_min_	1.50	1.22	1.75	1.25	1.57	1.32	1.34	1.41
*e*_max2_/*e*_min_	1.36	1.17	1.67	1.20	1.47	1.26	1.28	1.39
α_max1_	− 94°	+ 124°	− 92°	+ 125°	− 91°	+ 125°	+ 92°	− 125°
α_max2_	+ 92°	− 125^o^	+ 94°	+ 124°	+ 92°	− 125°	− 88°	+ 125°
α_min_	+ 1°	− 12^o^	+ 2°	− 8°	+ 3°	− 8°	+ 1°	+ 5°

*e*_max1_: primary maximum, *e*_max2_: secondary maximum, *e*_min_: minimum, α_max1_: azimuth angle of *e*_max1_, α_max2_: azimuth angle of *e*_max2_, α_min_: azimuth angle of *e*_min_.

From these results we conclude that if light energy absorbed by the inflorescence is a significant environmental factor for the development and maturation of seeds in sunflowers, then in Boone County the ideal azimuth direction of the inflorescence is almost exactly east. Such east-facing inflorescences absorb 10% and 50% more energy than those facing west and south, respectively.

According to Fig. 5B and Table 2, the back of a mature sunflower head absorbs maximal energy at α_max1_ =  + 124°, which is a northwest direction. The function *e*(α_n_) of the back has a secondary maximum at α_max2_ =  − 125° being a northeast direction. The minimum of *e*(α_n_) is at α_min_ =  − 12°, which is close to south. The back of a head whose inflorescence faces northwest absorbs *e*_max1_/*e*_max2_ = 1.04 and *e*_max1_/*e*_min_ = 1.22 times more energy than that of a head with inflorescence facing northeast and south (Table 2). Furthermore, the back of a head with inflorescence facing northeast instead of south absorbs *e*_max2_/*e*_min_ = 1.17 times more energy. These results suggest that in Boone County the head’s ideal azimuth angle would be α_n_ =  + 124° (northwest direction), if the maximization of energy absorbed by the back were the goal.

The above computations were repeated for the cloud conditions of central Italy (Fig. 2B), central Hungary (Fig. 2C), and south Sweden (Fig. 2D). For Italy and Hungary similar results were obtained as for Boone County (Fig. 5, Table 2): the most important finding is that the first maximum of *e*_inflorescence_(α_n_) is at α_n_ =  − 92° (Italy) and α_n_ =  − 91° (Hungary), while the first maximum of *e*_back_(α_n_) is at α_n_ =  + 125° (Italy and Hungary); furthermore, an east-facing sunflower inflorescence absorbs 5% (Italy) or 7% (Hungary) and 57% (Hungary) or 75% (Italy) more energy than that facing west and south, respectively. The reason for this similarity is that in Italy and Hungary the afternoons are also cloudier than the mornings, as in Bone County (Fig. 2A–C).

In contrast, in south Sweden mornings are cloudier than afternoons, that is σ_morning_(*t*) > σ_afternoon_(*t*) (Fig. 2D). The consequences of this are the following: a Swedish sunflower inflorescence absorbs maximal energy at α_max1_ =  + 92°, which is almost equal to west (α_west_ =  + 90°). The secondary maximum of *e*_inflorescence_(α_n_) is at α_max2_ =  − 88° being practically the same as east (α_east_ =  − 90°). The minimum of *e*_inflorescence_(α_n_) is at α_min_ =  + 1°, which is practically south (α_south_ = 0°). An inflorescence facing west absorbs 5% and 34% more energy than that facing east and south, respectively, while an inflorescence facing east instead of south absorbs 28% more energy.

In south Sweden the back of a mature sunflower head absorbs maximal energy at α_max1_ =  − 125°. The function *e*_back_(α_n_) has a secondary maximum at α_max2_ =  + 125° and a minimum at α_min_ =  + 5°. The back of a head with inflorescence facing northeast absorbs *e*_max1_/*e*_max2_ = 1.02 and *e*_max1_/*e*_min_ = 1.41 times more energy than that of a head with inflorescence facing northwest and south (Table 2). Furthermore, the back of a head with inflorescence facing northwest instead of south absorbs *e*_max2_/*e*_min_ = 1.39 times more energy.

From these we conclude that if the light energy absorbed by the inflorescence were the most important factor for mature sunflower heads, then in south Sweden the head’s ideal azimuth direction would be almost exactly west. On the other hand, in south Sweden the head’s ideal azimuth angle would be the northeast direction α_n_ =  − 125°, if the maximization of energy absorbed by the back were the aim.

According to Fig. 5, the energy absorbed by east-facing sunflower inflorescences decreases as the breeding area changes from Italy, through Hungary and Boone County to Sweden. The same is true for the energy absorbed by the back of mature sunflower heads, independently of their azimuth.

Our final conclusion is that the east facing of mature sunflower heads has the advantage of maximizing the total light energy absorbed by the inflorescence, if afternoons are cloudier than mornings as is the case in Boone County (as well as central Italy and Hungary). The excess energy is mainly due to the NUV, violet, blue and red spectral ranges in which the absorption of the inflorescence is large (Fig. 4A). This excess energy provides a plausible atmospheric-optical explanation as to why the eastward orientation of sunflower heads is the most advantageous under the asymmetric average daily cloudiness σ_morning_(*t*) < σ_afternoon_(*t*) in the area of the origin of cultivated sunflowers.

## Discussion

In sunflowers most of the light is intercepted by the leaves, with stem and petioles contributing less than 5% to total light absorption. The large amount of light received by the head increases the temperature of this organ and thus accelerates its development, especially the seed growth^27,28^. The existing six explanations for the east facing of mature sunflower heads mentioned in the Introduction are all hypothetical and untested. In order to reveal a potential new explanation of the ecological function of the permanent east facing of sunflower heads, in this work we computed the total light interception by the head after anthesis when solar tracking is stopped. We found that the energetically ideal azimuth direction for a mature sunflower inflorescence to receive maximal solar and sky radiation is east (Fig. 5).

Older sunflower heads leaning strongly downward can possess a curved surface, rather than a flat one. Such curved surfaces receive more direct sunlight than flat ones at lower solar elevations. Thus, our computations assuming a flat sunflower head slightly underestimate the total light energy received.

Because different regions have different diurnal cycle (and asymmetry) of cloudiness, the question arises: Why do mature sunflower heads orient always eastward regardless of geographic location? The answer might be due to the fact that domesticated sunflowers originate from eastern North America^26^, the cloudiness conditions of which are similar to those of Boone County. Thus, we performed our computations for Boone County, where afternoons are cloudier than mornings, the consequence of which is that sunflower inflorescences absorb maximal light energy if they face east. This ideal eastern orientation had been genetically coded in the domesticated *Helianthus annuus*, which later spread throughout the Earth. As a control situation, we demonstrated that if mornings are cloudier than afternoons, as is the case in South Sweden, then mature sunflower inflorescences absorb maximal energy, when they are oriented westward. Nevertheless, Swedish mature sunflower heads also face east, because during the relatively short period (a few hundred years) since their domestication they could not have adapted to the local cloudiness conditions. The genetic coding is the reason, we believe, for the observation that the eastward orientation of mature sunflower heads is independent of the specifics of regional cloudiness.

As shown in Table 2, the back of a mature sunflower head absorbs 4–5 times more energy than its inflorescence. The main reason for this is that the head’s elevation angle θ_n_ decreases steeply from 10° at anthesis to a minimum of − 75° on the 42nd day after anthesis (see Fig. 3). Thus, after the 2nd–3rd week past anthesis the inflorescence receives a considerably decreased light flux. The back of the head is green and later yellowish-green containing chlorophyll for photosynthesis (it is mainly the blue and red components of light that green plants require for their photosynthetic activity). If heads tended to maximize this photosynthesis, they should maximize the back-absorbed light energy, for which the ideal azimuth angle would be the northwest direction α_n_ =  + 124°. Since mature sunflower heads face east, this assumption is unsupported by observations. Consequently, the function of the green back of the head may be more akin to that of the green leaves: to take part in photosynthesis, independently of the head’s azimuth and elevation. Apart from the inflorescence, all aboveground parts of sunflowers contribute to the photosynthesis due to their green chlorophyll content.

However, we found that if afternoons are cloudier than mornings, then the total energy absorbed by the inflorescence is maximal at azimuth angle α_max_ =  − 94°, practically coinciding with the east direction (α_east_ =  − 90°). This can explain the east orientation of the mature heads of sunflowers originating from the region of Boone County, where afternoons are indeed cloudier than mornings in the growing season.

**Figure 6:**
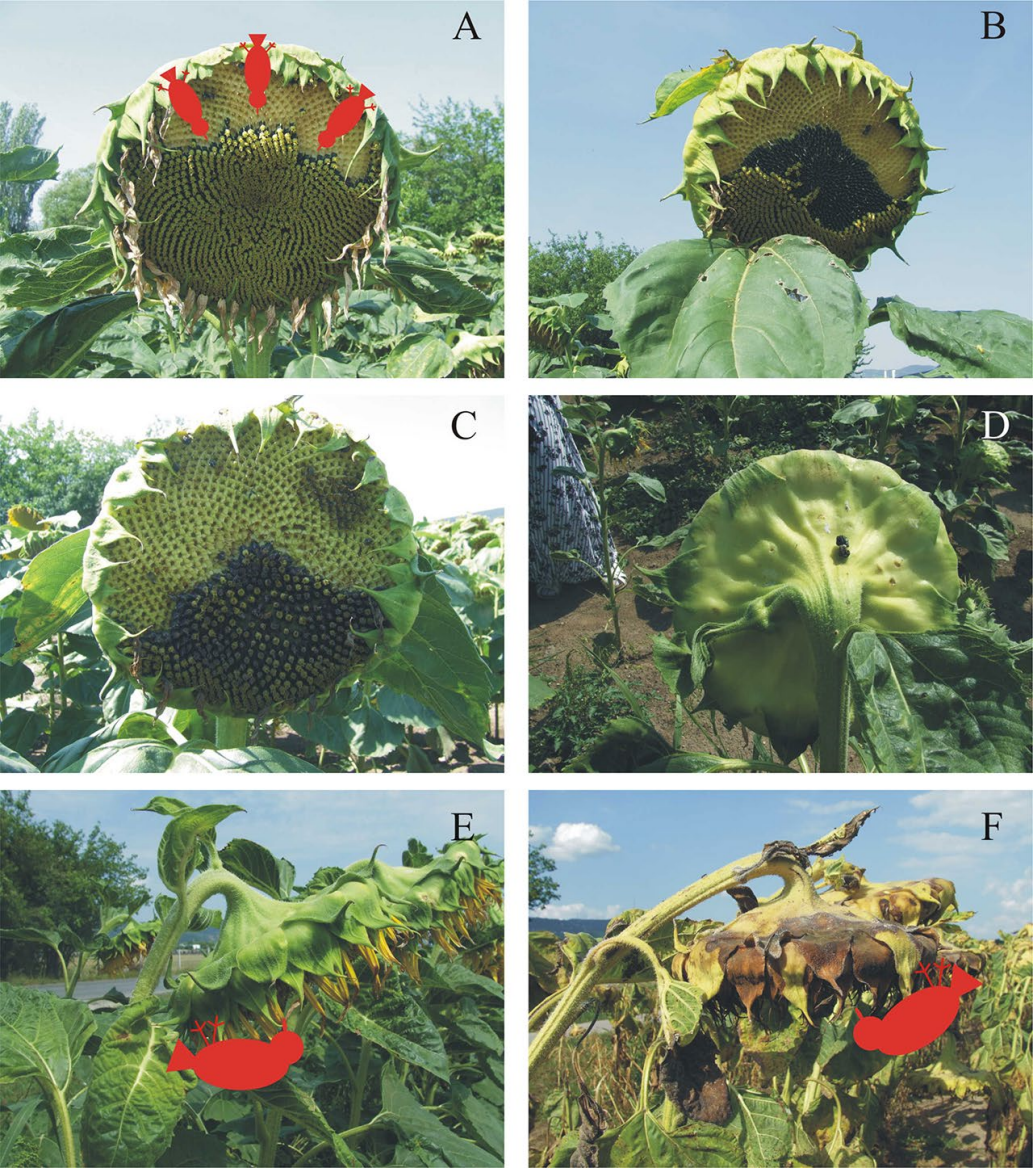
Bird-depredation of sunflower seeds. (**A**–**C**) The seeds of more elevated sunflower heads can be easier picked by birds (red schematic) clinging onto the head’s edge. (**D**) Dark excrement of a seed-eating bird on the back of a sunflower head. (**E**,**F**) The seeds of more downward-facing sunflower heads are more difficult for birds to reach. The yellow holes of picked-off dark grey seeds are clearly visible in pictures (**A**–**C**). Photos were taken by Gábor Horváth.

Let us briefly analyse the six hypotheses proposed earlier to explain the function(s) of east facing of sunflower heads:

1. According to Seiler^14^, a non-skyward orientation of the inflorescence of mature sunflower heads could reduce seed depredation by birds. This hypothesis is supported by our qualitative observation that birds clinging onto the edge of sunflower heads with higher elevation angles can easier pick the seeds than on more downward-facing heads. Birds usually land on the uppermost edge of the head, because they can pick the uppermost seeds the easiest (Fig. 6A–D). If the head is less elevated, birds can reach the downward-facing seeds with more difficulty (Fig. 6E,F). However, these observations are true for any azimuth direction of the head, thus the hypothesis of Seiler^14^ cannot explain the east facing of sunflower inflorescences.
2. According to the hypothesis of Leshem^15^ as well as Lang and Begg^4^, the easterly direction of sunflower inflorescences may have the advantage of reducing the heat load at noon. However, west-facing inflorescences would have the same advantage, furthermore, in this work we demonstrated that south-facing inflorescences absorb the least light energy (Fig. 5), which could considerably reduce the heat load at noon. Thus, this explanation for the east facing of sunflower inflorescences is unsupported by our calculations.
3. Lang and Begg^4^ hypothesized that the eastward orientation of sunflower inflorescences permits greater reception of solar radiation in the early morning, which may speed up the drying of morning dew and thereby could reduce the chance of fungal attack. This idea is supported by our finding that east-facing inflorescences absorb maximal light energy (Fig. 5). However, the assumed reduced fungal attack of east-facing inflorescences relative to west- or south-facing ones has to be tested in the future.
4. In the opinion of Lamprecht^16^, the eastward orientation of sunflower inflorescences may promote their attractiveness to pollinators through increased morning interception of solar radiation, coincident with the daily timing of anther emergence and pollen presentation. This hypothesis is also supported by our above-mentioned finding (Fig. 5). Nevertheless, the hypothesized larger attractiveness of east-facing inflorescences to pollinators in comparison with west/south-facing ones should still be tested in the field.
5. Seiler^14^ assumed that the eastward orientation of sunflower inflorescences could reduce heat load mainly in the afternoon periods of high irradiance. However, we showed here that the energy absorbed by east-facing inflorescences is larger than that absorbed by west- or south-facing inflorescences. Thus, this explanation is also contradicted by our calculations.
6. Ploschuk and Hall^18^ attempted to explain the east orientation of sunflower inflorescences by the lower head temperature which may be advantageous for seed maturation and grain filling. The head temperature is mainly determined by the sum of energies absorbed by both the inflorescence and the head’s back. According to Table 2, in Boone County, central Italy and Hungary, and south Sweden the minima of energies absorbed by the inflorescence and the back of sunflower heads combined are at nearly southern azimuth angles of α_min_ ≈ 0°. Consequently, this explanation seems to be unsound, too.

The 10–50% surplus energy absorbed by an east-facing sunflower inflorescence compared to an inflorescence facing west-south obtained in this work may be advantageous for seed development and maturation. Furthermore, this surplus energy could accelerate the evaporation of morning dew condensed on the inflorescence, leading to a reduced chance of fungal attack as hypothesized by Lang and Begg^4^. Another advantage of this surplus energy may be a stronger attraction of morning-active pollinators to east-facing inflorescences as hypothesized by Lamprecht^16^ and Atamian et al.^17^. Hence, our atmospheric-optical explanation for the merit of east facing of sunflower inflorescences is consistent with these two hypotheses.

**Figure 7:**
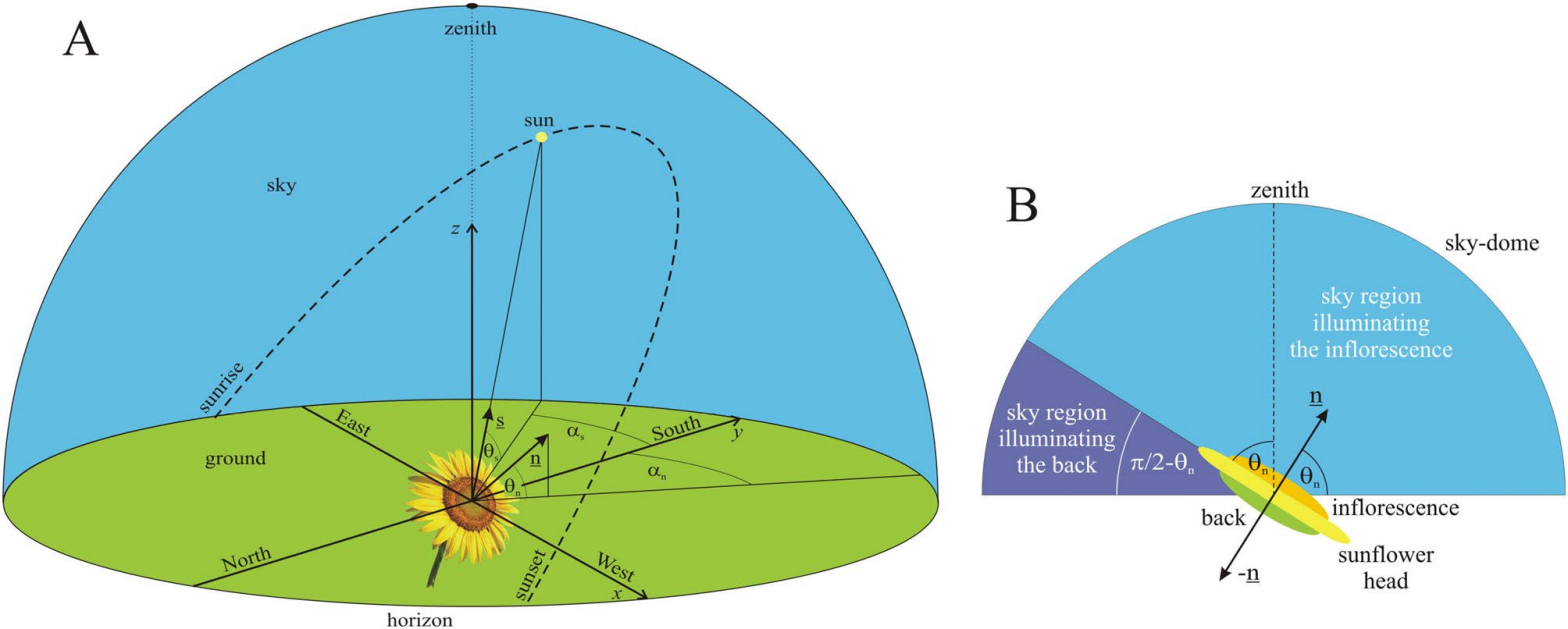
Geometry of a mature sunflower head and the sky. (**A**) Geometry of the sky-dome with the unit vectors n and s characterizing the normal vector of a mature sunflower head and the direction of the Sun, respectively. (**B**) For calculation of the proportion (θ_n_ + π/2)/π of the celestial hemisphere from which a sunflower inflorescence with elevation angle θ_n_ of its normal vector n receives diffuse skylight (light blue) and the proportion (π/2-θ_n_)/π of the sky-dome from which the back (with normal vector − n) of the head receives diffuse skylight (dark blue).

The ideal easterly azimuth direction of sunflower heads may be surprising, as one might expect south is ideal, given that solar panels are usually directed toward south. Although direct sunlight is the most intense at noon, it hits a tilted (θ_n_ <  + 40°, Fig. 3) sunflower head facing south at larger incident angles γ between the unit vectors n and s of the head and the sun rays (see Fig. 7A), and thus the received solar energy (being proportional to cosγ) is smaller. Although direct sunlight is less intense in the morning and afternoon than around noon, it hits a tilted (θ_n_ <  + 40°) sunflower head facing east or west at smaller γ, thus resulting in larger received solar energy. Under persistently clear skies the ideal east and west azimuth angles of sunflower heads would be mirror-symmetrical to south. However, this symmetry is broken if the frequency of clouds is larger in the afternoon than in the morning, as is usually the case in the region where sunflowers were domesticated (Fig. 2A). Under such an asymmetric diurnal cycle of cloudiness the energetically ideal azimuth direction of sunflower inflorescences is east (Fig. 5).

We found that depending on the regional cloud conditions, mature sunflower inflorescences facing east absorb 10% and 50% more radiation than those facing west and south, respectively, if the afternoons are cloudier than the mornings, as is typically the case in the cultivation regions of sunflowers. One might consider a 10% excess light energy insignificant. However, under the cloudy conditions typical for the emergence region of cultivated sunflowers this radiation excess can ecologically be advantageous, to which it is worth adapting. On the other hand, a 50% radiation excess is an obvious ecological advantage of east facing compared to south facing.

Finally, the question arises: If for mature sunflower inflorescences the energetically ideal azimuth direction is east, then why do solar panels usually face south? The resolution of this paradox is the following: (i) Sunflower heads absorb solar radiation to develop their seeds only for 1–2 months in the summer, while solar panels operate during the whole year. Per our explanation, one of the reasons for the east facing of sunflower heads is that easterly oriented inflorescences absorb maximal radiation because afternoons tend to be cloudier than mornings due to the well-known diurnal cycle of continental convection, which peaks in late afternoon. The strong asymmetry in daily cloudiness during the warmest and hence convectively most active summer months is lessened if averaged for the whole year. (ii) Depending on latitude, the normal vector of fixed solar panels has a relatively large positive elevation angle (20°–40°, the higher the latitude, the lower the angle), for which south facing maximizes the received sky radiation^29^. For mature sunflower inflorescences with elevation angles θ_n_ ≤  + 40° (Fig. 3) east azimuth maximizes the absorbed radiation as showed in this work.

## Materials and methods

### Calculation of solar elevation and azimuth angles versus time

For our numerical calculations, the solar elevation angle θ_s_(*t*) from the horizon and the solar azimuth angle α_s_(*t*) from south (axis *y*, Fig. 7A) were calculated as a function of time *t* with an algorithm based on a semi-analytical approximation (analytical Kepler’s orbits modified with astronomical perturbations) and the planetary theory VSOP 87 (Variations Séculaires des Orbites Planètaires) of Bretagnon and Francou^30^. This method is valid for the 1950–2050 period with an accuracy of 0.01°. Using this algorithm, we calculated the geocentric ecliptical, then the geocentric equatorial, and finally the geocentric horizontal coordinates of the Sun, resulting in the values of θ_s_(*t*) and α_s_(*t*).

### Diurnal cloudiness

Total cloud cover (TCC) time series of high temporal resolution (1 h) were evaluated for the period 01.01.2009–31.12.2018 from the ERA5 reanalysis of the European Centre for Medium-Range Weather Forecasts^31^. The geographic coverage is global with a native spatial resolution of 0.25° × 0.25° ≈ 27 km × 27 km. Climatological mean values of TCC were determined by averaging for each hour of each calendar day of every year in the vegetative period of sunflowers. Since TCC is a dimensionless relative parameter in the range 0–1 (0 is clear sky, 1 is overcast), the hourly climatological means are equivalent to the time-dependent probability 0 ≤ σ(*t*) ≤ 1 of cloudy situation. We determined the diurnal cloud probability function σ(*t*) in July, August and September in Boone County (Kentucky, USA, 39° N, − 84.75° E, Fig. 2A), central Italy (41.0° N, 15.0° E, Fig. 2B), central Hungary (47.0° N, 19.0° E, Fig. 2C), and south Sweden (58.0° North, 13.0° East, Fig. 2D). The cloudiness data used in our calculations correspond to the decade between 2009 and 2018. Because similar data are not readily available for the period when sunflowers were domesticated, we assume in this work that the data obtained in the last decade is historically representative. The validity of this assumption can be evaluated when paleo-climatological cloudiness data become available.

### Measurement of the elevation angle of mature sunflower heads versus time

In a sunflower plantation at Budaörs (near Budapest), we measured the elevation angle θ_n_ of the normal vector of the mature head of the same 100 sunflowers as a function of time *t*, approximately weakly from 6 July to 11 September 2020. The studied sunflowers were individuals in a given row of the plantation.

### Measurement of the absorption spectra of mature sunflower heads

The absorption spectra *A*(λ) of young (2 weeks after anthesis) and old (4 weeks after anthesis) inflorescence and back of mature sunflower heads were measured in the field with an Ocean Optics STS-VIS spectrometer (Ocean Insight, Largo, USA) in July 2020. Measurements were performed under total overcast conditions to ensure isotropic diffuse skylight illumination. At first, the reflection spectrum of the inflorescence/back was determined as follows: a spectrum was measured by directing the spectrometer’s head on the target at a distance of 5 cm, then another spectrum was registered by pointing the spectrometer to the overcast sky. In the laboratory these two spectra were divided by each other. Finally, assuming that all non-reflected light was absorbed, the absorption spectrum *A*(λ) = 1 − *R*(λ) was obtained by subtracting the reflection spectrum *R*(λ) from 1. Absorption spectra were measured for 3 sunflowers and then averaged.

### Calculation of sky irradiance absorbed by a sunflower inflorescence

In the *x*–*y*-*z* reference frame of Fig. 7A, let the normal vector of a mature sunflower inflorescence be

2 $$\underline {\text{n}} = \, \left( {{\text{cos}}\theta_{{\text{n}}} \cdot {\text{sin}}\alpha_{{\text{n}}} ,{\text{ cos}}\theta_{{\text{n}}} \cdot {\text{cos}}\alpha_{{\text{n}}} ,{\text{ sin}}\theta_{{\text{n}}} } \right),$$

where axes *x* and *y* point to west and south, axis *z* points vertically upward, the elevation angle − 90° ≤ θ_n_ ≤  + 90° is measured from the horizontal (θ_n_ > 0°: above the horizon, θ_n_ < 0°: below the horizon), and the azimuth angle α_n_ is measured clockwise from axis *y* (south). In our model, after sunflower anthesis angle α_n_ is constant, because the head does not follow the motion of the Sun in the sky, and angle θ_n_(*t*) decreases monotonously with time *t* (due to the gradually increasing weight of the head) as measured in the field (Fig. 3). The unit vector pointing toward the Sun is:

3 $$\underline {\text{s}} = \, \left( {{\text{cos}}\theta_{{\text{s}}} \cdot {\text{sin}}\alpha_{{\text{s}}} ,{\text{ cos}}\theta_{{\text{s}}} \cdot {\text{cos}}\alpha_{{\text{s}}} ,{\text{ sin}}\theta_{{\text{s}}} } \right),$$

where θ_s_ and α_s_ are the solar elevation and azimuth angles (Fig. 7A). When θ_n_ = 0° and θ_s_ = 0° the inflorescence is vertical and the Sun is on the horizon, while for θ_n_ = 90° and θ_s_ = 74.5° the horizontal inflorescence looks at the zenith and the Sun culminates on 21 June at the 39° northern latitude of Boone County (Kentucky, USA, 39° N, − 84.75° E), the region from which the domesticated sunflower originates^26^. In the case of α_n_ =  − 90°, 0° and 90°, the inflorescence faces east, south, and west, respectively. Northeast, southeast, southwest and northwest facing inflorescences are defined as − 180° < α_n_ <  − 90°, − 90° < α_n_ < 0°, 0° < α_n_ < 90° and 90° < α_n_ < 180°, respectively.

The global irradiance received from the sky-dome by a horizontal surface is the sum of the direct and diffuse irradiances:

4 $$I_{{{\text{global}}}} = I_{{{\text{direct}}}} + I_{{{\text{diffuse}}}} ,$$

and

5 $$I_{{{\text{diffuse}}}} = D \cdot I_{{{\text{global}}}} ,$$

where *D* is the diffuse fraction of global radiation. From (4) and (5) it follows:

6 $$I_{{{\text{diffuse}}}} = I_{{{\text{direct}}}} \frac{D}{1 - D}.$$

There are two different meteorological situations: (i) In cloudy situation with time-dependent probability 0 ≤ σ(*t*) ≤ 1 clouds dominate which frequently occlude the Sun. (ii) In sunny situation with probability 1 − σ(*t*) direct sunlight dominates, because the Sun is occluded by clouds only rarely. The total light energy absorbed by a sunflower inflorescence from dawn to dusk on the i-th day is the sum of the energy of direct solar radiation $E_{{\text{sun,i}}} ({\uptheta }_{{\text{n}}}, {{\alpha }}_{{\text{n}}} )$, the energy $E_{{\text{diffuse, i}}}^{{{\text{cloud}}y}} ({\uptheta }_{{\text{n}}}, {{\alpha }}_{{\text{n}}} )$ of diffuse radiation in cloudy weather, and the energy $E_{{\text{diffuse, i}}}^{{{\text{sunn}}y}} ({\uptheta }_{{\text{n}}}, {{\alpha }}_{{\text{n}}} )$ of diffuse radiation in sunny weather:

7 $$E_{{\text{i}}} = {E_{{\text{diffuse,i}}}^{{{\text{cloud}}y}}} ({\uptheta }_{{\text{n}}}, {{\alpha }}_{{\text{n}}} ) + {E_{{\text{sun,i}}}} ({\uptheta }_{{\text{n}}}, {{\alpha }}_{{\text{n}}} ) + {E_{{\text{diffuse,i}}}^{{{\text{sunn}}y}}} ({\uptheta }_{{\text{n}}} {,\alpha }_{{\text{n}}} ).$$

**Figure 8:**
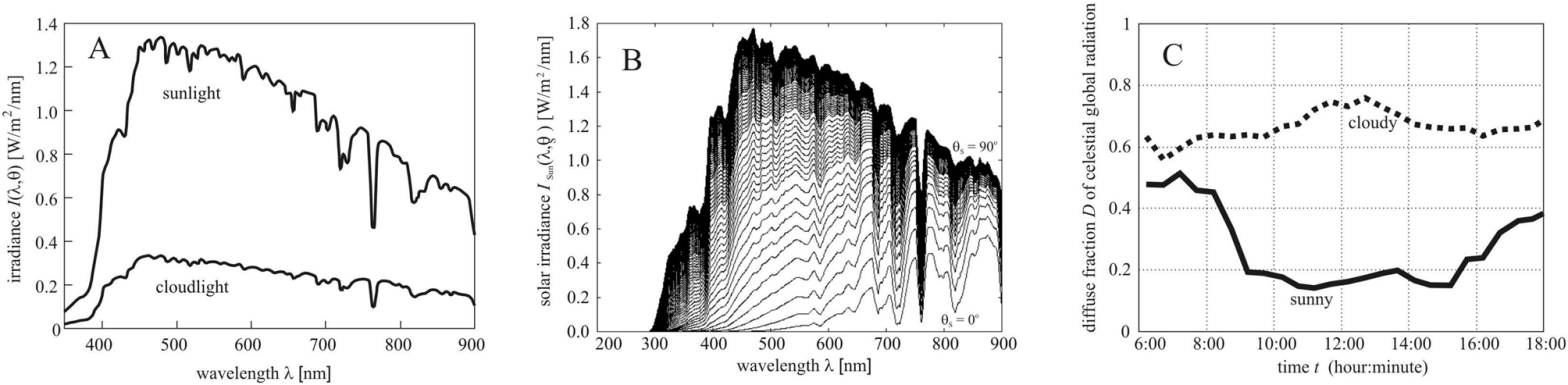
Meteorological data used in the computations. (**A**) Spectral distribution of irradiance *I*_sun_(λ, θ_s_) for direct sunlight measured under cloudless sky conditions at solar noon with solar elevation angle θ_s_ = 53.5° and *I*_cloud_(λ) for cloudlight measured on a completely overcast day at solar noon with solar elevation θ_s_ = 53.8° (after Fig. 2a of Dengel et al.^32^). (**B**) Spectral distribution of irradiance *I*_Sun_(λ, θ_s_) of direct sunlight as a function of solar elevation angle θ_s_ ranging from 0° (horizon, lowermost curve) to 90° (zenith, uppermost curve) with an increment of 1° simulated by MODTRAN 3.7 (Ref.^33^). (**C**) Diurnal variation of the annual mean diffuse fraction *D* (the proportion of diffuse sky radiation to global sky radiation) under sunny and cloudy conditions over northern China in April July 2017 (after Fig. 6 of Liu et al.^39^).

Under cloudy conditions the diffuse energy component is:

8 $${E_{{\text{diffuse,i}}}^{{{\text{cloud}}y}}} ({\uptheta }_{{\text{n}}}, {{\alpha }}_{{\text{n}}} ) = Q\frac{{{\uptheta }_{{\text{n}}} + {{\pi /2}}}}{{\uppi }}\int\limits_{{t_{{{\text{rise}}}}^{{\text{i}}} }}^{{t_{{{\text{set}}}}^{{\text{i}}} }} {\left\langle {{\sigma (}t{)}\int\limits_{{{\uplambda }_{{{\text{min}}}} }}^{{{\uplambda }_{{{\text{max}}}} }} {A_{{{\text{inflor}}}} ({\uplambda })I_{{{\text{diffuse}}}}^{{{\text{cloud}}y}} ({\lambda ,}t){\text{d}}\lambda } } \right\rangle {\text{d}}t} ,$$

where *Q* is the surface area of the sunflower inflorescence, $I_{{{\text{diffuse}}}}^{{{\text{cloud}}y}} ({{\lambda ,t}})$ is the diffuse irradiance received by a horizontal surface in cloudy situation, λ_min_ = 400 nm ≤ λ ≤ λ_max_ = 700 nm is the wavelength interval of radiation in which the light absorption by sunflowers is physiologically relevant^6^, $t_{{{\text{rise}}}}^{{\text{i}}}$ and $t_{{{\text{set}}}}^{{\text{i}}}$ denote the time of sunrise and sunset on the i-th day (after the first calendar day i = 1 the head does not follow the Sun), *A*_inflor_(λ) is the absorption spectrum of the inflorescence, and the factor (θ_n_ + π/2)/π describes the proportion of the sky hemisphere from which an inflorescence with elevation angle θ_n_ receives diffuse skylight (Fig. 7B). Figure 2A illustrates the average absorption spectra *A*_inflor_(λ) of young (2 weeks after anthesis) and old (4 weeks after anthesis) sunflower inflorescences. In our computations *A*_inflor_(λ) was set to the absorption spectrum of young inflorescences in the first 3 weeks after anthesis, and then it was set to that of old inflorescences. In the spectral range λ_min_ = 400 nm ≤ λ ≤ λ_max_ = 700 nm, under cloudy conditions the average direct radiation is approximately 20% of the direct solar radiation (Fig. 8A) and this fraction has only a little variation with sky condition and time of year^32^. Using (6) and the 0.2 factor, we obtain:

9 $$I_{{{\text{diffuse}}}}^{{{\text{cloud}}y}} ({{\uplambda ,t}}) = \frac{{D_{{{\text{cloudy}}}} (t)}}{{1 - D_{{{\text{cloudy}}}} (t)}}0.2I_{{{\text{sun}}}} [{\uplambda },{\uptheta }_{{\text{s}}}^{{\text{i}}} (t)]\sin {\uptheta }_{{\text{s}}}^{{\text{i}}} (t),$$

where *I*_sun_(λ, θ_s_) is the spectral distribution of irradiance of direct sunlight (Fig. 8B) incident on a unit surface perpendicular to the direct solar radiation, and *D*_cloudy_ is the diffuse fraction of global radiation under cloudy conditions (Fig. 8C). In meteorology, *I*_global_, *I*_direct_ and *I*_diffuse_ are always measured on a horizontal detector (radiometer) surface, while the irradiance *I*_Sun_(λ, θ_s_) of sunlight in Fig. 8B was computed for a surface being perpendicular to the direct solar radiation. In order to take into consideration these two different orientations of the detector surface, *I*_sun_(λ, θ_s_) is multiplied by sinθ_s_ in (9).

Using MODTRAN (MODerate resolution TRANsmittance code)^33–36^ version 3.7 with a solar constant of 1362.12 W/m^2^ and the 1976 US Standard Atmosphere^37^, we have computed the ground-level direct-normal spectral solar irradiance *I*_sun_(λ, θ_s_) as described by Egri et al.^38^. *I*_Sun_(λ, θ_s_) is the energy of solar radiation per unit time, per unit area perpendicular to the radiation and per unit wavelength interval. Figure 8B shows the irradiance spectrum *I*_Sun_(λ, θ_s_) of direct sunlight for 91 different solar elevation angles θ_s_.

Figure 8C shows the diurnal variation of the annual mean diffuse fraction *D* under cloudy and sunny conditions measured by Liu et al.^39^ over northern China. Since we did not find similar publicly available measurements for other regions of the Earth, we use these data further on in this work. From (8) and (9) we obtain the diffuse light energy per unit area received by a sunflower inflorescence under cloudy conditions:

10 $$e_{{\text{diffuse,i}}}^{{{\text{cloud}}y}} ({\uptheta }_{{\text{n}}} {{,\alpha }}_{{\text{n}}} ) = \frac{{E_{{\text{diffuse,i}}}^{{{\text{cloud}}y}} ({\uptheta }_{{\text{n}}} {{,\alpha }}_{{\text{n}}} )}}{Q} = 0.2\frac{{{\uptheta }_{{\text{n}}} + {{\pi /2}}}}{{\uppi }}\int\limits_{{t_{{{\text{rise}}}}^{{\text{i}}} }}^{{t_{{{\text{set}}}}^{{\text{i}}} }} {\left\langle {\frac{{{\sigma (}t{)}D_{{{\text{cloudy}}}} (t)\sin {\uptheta }_{{\text{s}}}^{{\text{i}}} (t)}}{{1 - D_{{{\text{cloudy}}}} (t)}}\int\limits_{{{\uplambda }_{{{\text{min}}}} }}^{{{\uplambda }_{{{\text{max}}}} }} {A_{{{\text{inflor}}}} ({\uplambda })I_{{{\text{sun}}}} [{\uplambda },{\uptheta }_{{\text{s}}}^{{\text{i}}} (t)]{{d}}\lambda } } \right\rangle {\text{d}}t} .$$

If *I*_direct_(λ, θ_s_) is the direct irradiance illuminating the sunflower [Joule/(second·nanometer·meter^2^)], then the elementary direct light energy d*E* absorbed by the inflorescence in a time interval d*t* and in a wavelength bin dλ is:

11 $${\text{d}}E = Q \cdot {\text{cos}}\gamma \cdot I_{{{\text{direct}}}} \left( {\lambda , \, \theta_{{\text{s}}} } \right) \cdot A_{{{\text{inflor}}}} \left( \lambda \right) \cdot {\text{d}}\lambda \cdot {\text{d}}t,$$

where γ is the incidence angle between the unit vectors n (n = 1) and s (s = 1), the cosine of which can be expressed as:

12 $${\text{cos}}\gamma \, = {\underline{\text{n}}} \cdot {\underline{\text{s}}} = {\text{ cos}}\theta_{{\text{n}}} \cdot {\text{sin}}\alpha_{{\text{n}}} \cdot {\text{cos}}\theta_{{\text{s}}} \cdot {\text{sin}}\alpha_{{\text{s}}} + {\text{ cos}}\theta_{{\text{n}}} \cdot {\text{cos}}\alpha_{{\text{n}}} \cdot {\text{cos}}\theta_{{\text{s}}} \cdot {\text{cos}}\alpha_{{\text{s}}} + {\text{ sin}}\theta_{{\text{n}}} \cdot {\text{sin}}\theta_{{\text{s}}} .$$

The inflorescence can absorb direct sunlight only if the following condition is satisfied:

 − 90° < γ <  + 90°, 0 < cosγ < 1, that is

13 $$0 < {\text{cos}}\theta_{{\text{n}}} \cdot {\text{sin}}\alpha_{{\text{n}}} \cdot {\text{cos}}\theta_{{\text{s}}} \cdot{\text{sin}}\alpha_{{\text{s}}} + {\text{ cos}}\theta_{{\text{n}}} \cdot{\text{cos}}\alpha_{{\text{n}}} \cdot {\text{cos}}\theta_{{\text{s}}} \cdot {\text{cos}}\alpha_{{\text{s}}} + {\text{ sin}}\theta_{{\text{n}}} \cdot {\text{sin}}\theta_{{\text{s}}} < { 1}.$$

Using (11), under sunny conditions with probability 1 – σ(*t*), the direct solar energy per unit area received by the inflorescence is:

14 $$e_{{\text{sun,i}}} ({\uptheta }_{{\text{n}}} {{,\alpha }}_{{\text{n}}} ) = \frac{{E_{{\text{sun,i}}} ({\uptheta }_{{\text{n}}} {{,\alpha }}_{{\text{n}}} )}}{Q} = \int\limits_{{t_{{{\text{rise}}}}^{{\text{i}}} }}^{{t_{{{\text{set}}}}^{{\text{i}}} }} {\left\langle {[1 - {\sigma (}t{)]}\cos {\gamma (}t{)}\int\limits_{{{\uplambda }_{{{\text{min}}}} }}^{{{\uplambda }_{{{\text{max}}}} }} {A_{{{\text{inflor}}}} ({\uplambda })I_{{{\text{sun}}}} [{\uplambda },{\uptheta }_{{\text{s}}}^{{\text{i}}} (t)]{\text{d}}\lambda } } \right\rangle {\text{d}}t} ,$$

where the factor

15 $$\cos {\gamma (}t{)} = \cos {\uptheta }_{{\text{n}}} \sin {\upalpha }_{{\text{n}}} \cos {\uptheta }_{{\text{s}}}^{{\text{i}}} (t)\sin {\upalpha }_{{\text{s}}}^{{\text{i}}} (t) + \cos {\uptheta }_{{\text{n}}} \cos {\upalpha }_{{\text{n}}} \cos {\uptheta }_{{\text{s}}}^{{\text{i}}} (t)\cos {\upalpha }_{{\text{s}}}^{{\text{i}}} (t) + \sin {\uptheta }_{{\text{n}}} \sin {\uptheta }_{{\text{s}}}^{{\text{i}}} (t),$$

is necessary, because the direct solar radiation is usually not perpendicular to the surface of the inflorescence. Using (6) under sunny conditions, the diffuse irradiance received by a horizontal surface is:

16 $$I_{{{\text{diffuse}}}}^{{{\text{sunny}}}} ({{\lambda ,t}}) = \frac{{D_{{{\text{sunny}}}} (t)}}{{1 - D_{{{\text{sunny}}}} (t)}}I_{{{\text{sun}}}} [{\uplambda },{{\uptheta }_{{\text{s}}}^{{\text{i}}}} (t)]\sin {\uptheta }_{{\text{s}}}^{{\text{i}}} (t),$$

where the factor sinθ_s_ is again necessary for the reason mentioned when (9) was derived. Figure 8C shows the diurnal variation of the annual mean diffuse fraction *D*_sunny_ under sunny conditions^39^. Using (16), under sunny conditions with probability 1 − σ(*t*), the diffuse light energy per unit area received by the inflorescence is:

17 $$e_{{\text{diffuse,i}}}^{{{\text{sunn}}y}} ({\uptheta }_{{\text{n}}} {{,\alpha }}_{{\text{n}}} ) = \frac{{E_{{\text{diffuse,i}}}^{{{\text{sunn}}y}} ({\uptheta }_{{\text{n}}} {{,\alpha }}_{{\text{n}}} )}}{Q} = \frac{{{\uptheta }_{{\text{n}}} + {{\pi /2}}}}{{\uppi }}\int\limits_{{t_{{{\text{rise}}}}^{{\text{i}}} }}^{{t_{{{\text{set}}}}^{{\text{i}}} }} {\left\langle {\frac{{[1 - {\sigma (}t{)]}D_{{{\text{sunny}}}} (t)\sin {\uptheta }_{{\text{s}}}^{{\text{i}}} (t)}}{{1 - D_{{{\text{sunny}}}} (t)}}\int\limits_{{{\uplambda }_{{{\text{min}}}} }}^{{{\uplambda }_{{{\text{max}}}} }} {A_{{{\text{inflor}}}} ({\uplambda })I_{{{\text{sun}}}} [{\uplambda },{\uptheta }_{{\text{s}}}^{{\text{i}}} (t)]{\text{d}}\lambda } } \right\rangle {\text{d}}t} ,$$

where the factor (θ_n_ + π/2)/π is again the proportion of the sky hemisphere from which the inflorescence receives diffuse skylight (Fig. 7B). Finally, the total light energy *e*_total_ per unit area absorbed by a mature sunflower inflorescence in the period between the stop of solar tracking and senescence is:

18 $$e_{{{\text{total}}}} = \sum\limits_{{{\text{i}} = 1}}^{{{\text{i}} = {\text{m}}}} {e_{{\text{i}}} } = \sum\limits_{{{\text{i}} = 1}}^{{{\text{i}} = {\text{m}}}} {\left[ {e_{{\text{diffuse,i}}}^{{{\text{cloud}}y}} ({\uptheta }_{{\text{n}}} {{,\alpha }}_{{\text{n}}} ) + e_{{\text{sun,i}}} ({\uptheta }_{{\text{n}}} {{,\alpha }}_{{\text{n}}} ) + e_{{\text{diffuse,i}}}^{{{\text{sunn}}y}} ({\uptheta }_{{\text{n}}} {{,\alpha }}_{{\text{n}}} )} \right]} ,$$

where *m* is the last day of senescence, when the seeds are fully developed and ripe.

### Calculation of sky irradiance absorbed by the back of sunflower heads

The back side of sunflower heads is green (later yellowish green) due to its chlorophyll content, because it takes an important role in photosynthesis^6^. Thus, we calculate here the total light energy *e*_total,back_ per unit area absorbed by the back of a mature sunflower head in the period between anthesis and senescence. The back receives the same three radiation components as the inflorescence. The only three differences are the following: (i) The normal vector n_back_ of the back is opposite to the normal vector n of the inflorescence (Fig. 7B): n_back_ = −n. (ii) The back is illuminated by diffuse light originating from the sky region which is the complementary part of the sky-dome illuminating diffusely the inflorescence. This can be taken into consideration by factor 1 − [θ_n_(*t*) + π/2]/π = [π-2θ_n_(*t*)]/(2π), being the proportion of the sky from which the back of a sunflower head with elevation angle θ_n_(*t*) receives diffuse skylight (Fig. 7B). (iii) The absorption spectrum *A*_back_(λ) of the back of sunflower heads is different from the absorption spectrum *A*_inflor_(λ) of the inflorescence (Fig. 2). Taking into account these differences, we obtain the total light energy *e*_total,back_ per unit area absorbed by the back of a mature sunflower head in the period between anthesis and senescence as follows:

19 $$e_{{\text{total,back}}} = \sum\limits_{{{\text{i}} = 1}}^{{{\text{i}} = {\text{m}}}} {\left\{ {e_{{\text{diffuse,i,back}}}^{{{\text{sunn}}y}} \left[ {{\uptheta }_{{\text{n}}} {(}t{{),\alpha }}_{{\text{n}}} } \right] + e_{{\text{diffuse,i,back}}}^{{{\text{cloud}}y}} \left[ {{\uptheta }_{{\text{n}}} {(}t{{),\alpha }}_{{\text{n}}} } \right] + e_{{\text{sun,i,back}}} \left[ {{\uptheta }_{{\text{n}}} {(}t{{),\alpha }}_{{\text{n}}} } \right]} \right\}} ,$$

where *m* is the last day of senescence, and the three components in sum (19) are:

20 $$e_{{\text{diffuse,i,back}}}^{{{\text{sunn}}y}} [{\uptheta }_{{\text{n}}} (t){{,\alpha }}_{{\text{n}}} ] = \frac{{{\uppi } - {{2\uptheta }}_{{\text{n}}} (t)}}{{{{2\uppi }}}}\int\limits_{{t_{{{\text{rise}}}}^{{\text{i}}} }}^{{t_{{{\text{set}}}}^{{\text{i}}} }} {\left\langle {\frac{{\left[ {1 - {\sigma (}t{)}} \right]D_{{{\text{sunny}}}} (t)\sin {\uptheta }_{{\text{s}}}^{{\text{i}}} (t)}}{{1 - D_{{{\text{sunny}}}} (t)}}\int\limits_{{{\uplambda }_{{{\text{min}}}} }}^{{{\uplambda }_{{{\text{max}}}} }} {A_{{{\text{back}}}} ({\uplambda })I_{{{\text{sun}}}} [{\uplambda },{\uptheta }_{{\text{s}}}^{{\text{i}}} (t)]{\text{d}}\lambda } } \right\rangle {\text{d}}t} ,$$

21 $${e_{{\text{diffuse,i,back}}}^{{{\text{cloud}}y}}} [{\uptheta }_{{\text{n}}} (t){{,\alpha }}_{{\text{n}}} ] = 0.2\frac{{{\uppi } - {{2\theta }}_{{\text{n}}} (t)}}{{{{2\pi }}}}\int\limits_{{t_{{{\text{rise}}}}^{{\text{i}}} }}^{{t_{{{\text{set}}}}^{{\text{i}}} }} {\left\langle {\frac{{{\sigma (}t{)}D_{{{\text{cloudy}}}} (t)\sin {\uptheta }_{{\text{s}}}^{{\text{i}}} (t)}}{{1 - D_{{{\text{cloudy}}}} (t)}}\int\limits_{{{\uplambda }_{{{\text{min}}}} }}^{{{\uplambda }_{{{\text{max}}}} }} {A_{{{\text{back}}}} ({\uplambda })I_{{{\text{sun}}}} [{\uplambda },{\uptheta }_{{\text{s}}}^{{\text{i}}} (t)]{\text{d}}\lambda } } \right\rangle {\text{d}}t} ,$$

22 $$e_{{\text{sun,i,back}}} [{\uptheta }_{{\text{n}}} (t){{,\alpha }}_{{\text{n}}} ] = \int\limits_{{t_{{{\text{rise}}}}^{{\text{i}}} }}^{{t_{{{\text{set}}}}^{{\text{i}}} }} {\left\langle {\left[ {1 - {\sigma (}t{)}} \right] \cdot \left[ { - \cos {\gamma (}t{)}} \right]\int\limits_{{{\uplambda }_{{{\text{min}}}} }}^{{{\uplambda }_{{{\text{max}}}} }} {A_{{{\text{back}}}} ({\uplambda })I_{{{\text{sun}}}} [{\uplambda },{\uptheta }_{{\text{s}}}^{{\text{i}}} (t)]{\text{d}}\lambda } } \right\rangle {\text{d}}t} .$$

The component *e*_sun,i,back_[θ_n_(*t*),α_n_] expressed by (22) increases only when the back absorbs direct sunlight, which happens only, if the following condition is satisfied:

23 $$- 1 < \cos {\gamma (}t{)} = \cos {\uptheta }_{{\text{n}}} \sin {\upalpha }_{{\text{n}}} \cos {\uptheta }_{{\text{s}}}^{{\text{i}}} (t)\sin {\upalpha }_{{\text{s}}}^{{\text{i}}} (t) + \cos {\uptheta }_{{\text{n}}} \cos {\upalpha }_{{\text{n}}} \cos {\uptheta }_{{\text{s}}}^{{\text{i}}} (t)\cos {\upalpha }_{{\text{s}}}^{{\text{i}}} (t) + \sin {\uptheta }_{{\text{n}}} \sin {\uptheta }_{{\text{s}}}^{{\text{i}}} (t) < 0.$$

Since in this case cosγ(*t*) < 0, the negative sign of cosγ(*t*) in (22) is necessary.

Figure 2B shows the average absorption spectra *A*_back_(λ) of young (2 weeks after anthesis) and old (4 weeks after anthesis) backs of sunflower heads. In our computations *A*_back_(λ) was set to the absorption spectrum of young backs in the first 3 weeks after anthesis, and then it was set to that of old backs.

### Ethical approval and informed consent

For our studies no permission, licence or approval was necessary.

## Data Availability

Our paper has no electronic supporting material.
